# Screening Acute HIV Infections among Chinese Men Who Have Sex with Men from Voluntary Counseling & Testing Centers

**DOI:** 10.1371/journal.pone.0028792

**Published:** 2011-12-14

**Authors:** Xiaoxu Han, Junjie Xu, Zhenxing Chu, Di Dai, Chunming Lu, Xu Wang, Li Zhao, Cheng Zhang, Yangtao Ji, Hui Zhang, Hong Shang

**Affiliations:** 1 Key Laboratory of AIDS Immunology of Ministry of Health, Department of Laboratory Medicine, No.1 Hospital of China Medical University, Shenyang, Liaoning, China; 2 HIV and Sexually Transmitted Disease Prevention and Control Institute, Liaoning Center for Disease Control and Prevention, Shenyang, Liaoning, China; 3 HIV and Sexually Transmitted Disease Prevention and Control Department, Fushun Center for Disease Control and Prevention, Fushun, Liaoning, China; 4 HIV and Sexually Transmitted Disease Prevention and Control Department, Anshan Center for Disease Control and Prevention, Anshan, Liaoning, China; New York Blood Center, United States of America

## Abstract

**Background:**

Recent studies have shown the public health importance of identifying acute HIV infection (AHI) in the men who have sex with men (MSM) of China, which has a much higher risk of HIV transmission. However, cost-utility analyses to guide policy around AHI screening are lacking.

**Methodology/Principal Findings:**

An open prospective cohort was recruited among MSM living in Liaoning Province, Northeast China. Blood samples and epidemiological information were collected every 10 weeks. Third-generation ELISA and rapid test were used for HIV antibody screening, western blot assay (WB) served for assay validation. Antibody negative specimens were tested with 24 mini-pool nucleic acid amplification testing (NAAT). Specimens with positive ELISA but negative or indeterminate WB results were tested with NAAT individually without mixing. A cost-utility analysis of NAAT screening was assessed. Among the 5,344 follow-up visits of 1,765 MSM in 22 months, HIV antibody tests detected 114 HIV chronic infections, 24 seroconverters and 21 antibody indeterminate cases. 29 acute HIV infections were detected with NAAT from 21 antibody indeterminate and 1,606 antibody negative cases. The HIV-1 prevalence and incidence density were 6.6% (95% CI: 5.5–7.9) and 7.1 (95% CI: 5.4–9.2)/100 person-years, respectively. With pooled NAAT and individual NAAT strategy, the cost of an HIV transmission averted was $1,480. The addition of NAAT after HIV antibody tests had a cost-utility ratio of $3,366 per gained quality-adjusted life year (QALY). The input-output ratio of NAAT was about 1∶16.9.

**Conclusions/Significance:**

The HIV infections among MSM continue to rise at alarming rates. Despite the rising cost, adding pooled NAAT to the HIV antibody screening significantly increases the identification of acute HIV infections in MSM. Early treatment and target-oriented publicity and education programs can be strengthened to decrease the risk of HIV transmission and to save medical resources in the long run.

## Introduction

Recent data indicate that human immunodeficiency virus (HIV) infections have been rising sharply among the men who have sex with men (MSM) high-risk population in China [1], [2], [3], [4], [5]. In a recent large-scale national survey conducted in 2008 across 61 cities throughout China, covering over 18,000 MSM, the prevalence of human immunodeficiency virus (HIV) was 4.9% and incidence ranged from 2.6 to 5.4 per 100 person-years [6]. MSM is becoming one of the most important target populations for HIV prevention in China. The increasing trends of HIV infection in the MSM population also reported in the neighboring countries, such as Thailand [7], Myanmar [8] India [9] and Russia [10]. Liaoning Province is an important industrial province located in Northeast China with an estimated population of approximately 43 million people, among which 400,000 are MSM [11]. The MSM population in Liaoning is sexually active, often with multiple sex partners and occasional sex partners [1], [12]. The HIV prevalence in Liaoning MSM is increasing rapidly, as demonstrated by a recent prospective cohort study reporting the HIV incidence density of Shenyang, the capital of Liaoning, to reach 5.4/100 person-years [1]. Early detection of acutely infected HIV cases will allow behavioral interventions to become more readily available to patients, thereby preventing second-generation transmission through high-risk behavior reduction. Furthermore, the HIV infected individual can receive earlier treatment and improve his or her quality of life while decreasing infectivity [13], [14].

Third-generation enzyme-linked immunosorbent assay (ELISA) plus western blot were routinely used in HIV infection screening in both develpoed and developing countries in the world [15], [16], [17]. Since routine HIV antibody tests yield negative results during the first several weeks prior to seroconversion, acute infections can be diagnosed during this window period only with the use of tests for viral antigens and nucleic acids [18], [19]. In many developed countries, pooled NAAT were routinely used to detect the acute HIV infection (AHI) in antibody negative blood donation samples to reduce the residual risk of HIV transmission during the seronegative window period [20], [21], [22], [23]. Reports from developed countries showed limited marginal value to NAAT blood screening in improving blood safety [20], [21], [24], because the HIV prevalence was rather low and the number of donors in the window period at the time of donation was generally very low in those countries. With regards to the cost-effectiveness of pooled NAAT to detect AHI in the general population and high-risk sub-populations, some studies reported that pooled NAAT screening for AHI was not likely to be cost-effective in most settings, except for high-risk persons with very high incidences [25], [26], [27]. Although pooled NAAT can identify a substantial increased proportion of AHI at high risk for further HIV transmission [28], there has been no systematical research on the cost-effectiveness of AHI screening through HIV-1 antibody test and pooled NAAT in a high-risk population with high HIV incidence, especially in a cohort study.

In this study, we recruited a large-scale open prospective cohort of high-risk MSM from Voluntary Counseling and Testing (VCT) centers in three cities of Liaoning Province: Shenyang, Anshan and Fushun. This is the first large-scale prospective cohort in China utilizing third-generation ELISA antibody test and pooled NAAT for HIV screening. We aim to elucidate the HIV prevalence and incidence in this MSM high-risk population as well as analyze the cost-utility of pooled NAAT HIV testing on identifying acute HIV infections. This study is helpful for further investigating the newest trends of HIV infection in the MSM high-risk population and for deciding upon scientific control and medical resource configuration strategies.

## Results

### Cohort Follow-up

Between February 2009 and September 2010, 1765 MSM from Liaoning Province who met the inclusion criteria were recruited. In total, there were 5400 person times follow-up in the observation period, among which, 5344 person times follow-up were effective. 56 person times follow-up were excluded due to insufficient data. Among the 1765 cases, 1056 (59.8%) were followed-up for two or more times.

### HIV-1 Infection Identification

**Figure 1:**
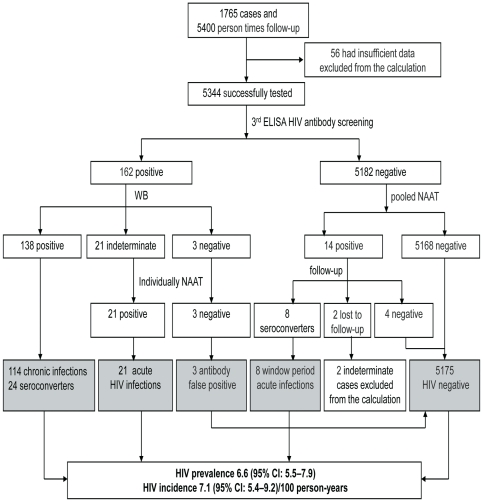
Flow Chart of the Study MSM High-risk Population and HIV Infection Detection Process. The numbers of study samples, HIV detection assays and the number of different results are shown in the flow chart. The different HIV statuses are indicated in the shaded areas. HIV prevalence and incidence are calculated based on the numbers in the shaded areas.

Among the 5344 blood samples, 5182 were negative and 162 were positive in the third-generation ELISA and the rapid HIV antibody test. After confirming with WB, 138 met the HIV-positive standard, including 114 baseline samples and 24 follow-up seroconversion samples. The latter were diagnosed as recent HIV infections. There were 21 samples with ELISA positive but WB indeterminate results, including 3 baseline samples and 18 follow-up samples. The 21 cases were all positive in NAAT and were diagnosed acute HIV infections due to an increase of bands in their follow-up samples confirmed with WB. Another 3 samples with positive ELISA but negative WB results were all negative in NAAT. No changes in HIV specific bands were detected in follow-up samples; therefore, these 3 cases were considered to be false positives for HIV antibody screening. To find serological window period HIV infected cases, 5182 antibody negative samples were mixed according to the 24 mini-pool strategy. Among them, 14 NAAT positive samples were detected. After follow-up, 8 cases that became seropositive were considered to be window period acute HIV infections, while another 4 cases that remained seronegative were considered to be false positives of the pooled NAAT. The remaining two cases were lost to follow-up, whose HIV statuses could not be confirmed thus were excluded from the following calculation. According to the aforementioned results and the epidemiology surveys, 114 chronic HIV infections and 53 acute or recent HIV infections were found. The latter included 24 seroconversion cases, 21 antibody positive/WB indeterminate/NAAT positive cases, as well as 8 serological window period cases. Overall, the calculated HIV prevalence and incidence in the MSM population in Liaoning were 6.6 (95% CI: 5.5–7.9) and 7.1 (95% CI: 5.4–9.2)/100 person-years, respectively (see Figure 1).

### Validity and predictive effect of the third-generation ELISA and NAAT

In this study, the golden standard consisted of a positive ELISA confirmed with western blot assay in the observation period. The sensitivity and specificity of the third-generation ELISA HIV antibody test for this MSM high risk population were 95.21%(95%CI: 90.78–97.91%) and 99.95%(95%CI: 99.83–99.99%), respectively. The positive predictive value of the third-generation ELISA HIV antibody test on this MSM high risk population was 98.15% (95%CI: (94.68–99.62%)). The sensitivity and specificity of pooled NAAT were 100% and 99.92%(95%: 99.80–99.98%), respectively. The positive predictive value of the pooled and individual NAAT after serological assay was 87.88% (95%CI, 71.80–96.60%) (Table 1). As a result, combining pooled or individual NAAT with third-generation ELISA, the sensitivity increased by 4.79% at the cost of 0.03% drop in specificity.

**Table 1:** Performance of 3^rd^ ELISA and NAAT on HIV Diagnosis in MSM High-risk Population.

Diagnostic Test	Results	HIV Infected Subjects (n)	Uninfected Subjects (n)	Total (n)	Sensitivity % (95%CI)	Specificity % (95%CI)	positive predictive value % (95%CI)
3^rd^ ELISA	Positive	159	3	162	95.21% (90.78–97.91%)	99.95% (99.83–99.99%)	98.15% (94.68–99.62%)
	Negative	8	5174	5182			
	Total	167	5177	5344			
Pooled/Individual NAAT	Positive	29	4	33	100%	99.92% (99.80–99.98%)	87.88% (71.80–96.60%)
	Negative	0	5171	5171			
	Total	29	5175	5204			

### Cost-effectiveness of Early Identification of Acute Infection with NAAT

With the HIV antibody assay, the cost was $3 for each specimen,$114 for each HIV infection, and $656 for each recent HIV infection identified. The addition of pooled NAAT after a third-generation ELISA to detect AHI, resulted in additional cost of $5.6 per specimen, $998 per acute HIV infection, and $3618 per window period acute HIV infection identified averagely. However, the addition of NAAT was estimated to avert 19.56 HIV transmissions, according to the formula in the methods. The cost of each HIV transmission averted was $1480. Based on the first-line antiretroviral regimen and monitoring programme currently used in China, the expense on treatment, CD4 count, and viral load monitoring was estimated to be $781. The additional life expectancy for HIV-1 patients treated with HAART was 32 years. The total cost of treatment and monitoring for each transmitted HIV infection within the life expectancy was $25000. The total averted treatment and monitoring expense was $489012. This means $460070 would be saved at the cost of $28942 on NAAT. The input-output ratio was 1∶16.9. Based upon the average life span for Chinese males, 7 QALYs were lost due to HIV infection for each patient in the HAART era and 136.9 QALYs lost can be averted in total. Therefore, the addition of pooled NAAT after third-generation ELISA to detect AHI had a cost-utility ratio of $3360 per gained QALY.

## Discussion

ELISA is the screening test that is commonly used for detection of HIV antibodies. Since it was first introduced in 1985, numerous commercial ELISA assays have been developed. Most of the first generation ELISA tests used viral lysate antigens that frequently had nonspecific reactions between the antibodies and the cell antigens. The second generation ELISA assays used recombinant proteins and/or synthetic peptides. However, these assays usually employed a conserved region of the HIV proteins and many of them failed to detect highly divergent HIV subtypes. The earlier generations of ELISAs usually had a 6 to 12 weeks window period. Targeting both IgM and IgG, the third-generation ELISA assays shorten the window period to about 3 weeks [18], [29], [30]. This window period can be further shortened to about 2 weeks with 4th generation ELISA that simultaneously detect both antigen and antibody [26]. Until recently, third-generation ELISA HIV antibody assay was still the fundamental tool for HIV detection in most hospitals and disease prevention and control centers in China. However, in this study, we found that the third generation ELISA HIV antibody assay can only correctly detect 95.21% of HIV infections in the MSM high-risk population, which is significantly lower than its performance for low risk populations. The main reason for the relatively low sensitivity is because the HIV incidence of MSM population was as high as 7.1/100 person-years. Some acute HIV infectioned cases were still seronegative or the antibody titer had not reach the lowest detection limit of the third-generation ELISA. So, third-generation ELISA is not enough for acute HIV infection screening in populations with high HIV incidence.

In the early stage of HIV-1 infection, the viremia arises before the production of antigen and antibody. Therefore, theoretically, NAAT can detect the evidence of HIV infection earlier. In this study, all antibody negative samples were mixed into 24 mini-pools for HIV NAAT, the low detection limit for the first level pools was as low as 40 copies/ml, the low detection limit for each sample was 960 copies/ml theoretically,, far below the general level of acute HIV infection [31]. 8 serological window period acute HIV infections were detected and confirmed. The sensitivities of third-generation ELISA and pooled NAAT were 95.21% and 100%, respectively. Therefore, in the MSM high risk population, the sensitivity increased greatly by combining NAAT with third-generation ELISA. It is important to note that the positive predictive value of the pooled and individual NAAT after serological assay was only 87.88%. Because of the high sensitivity of NAAT, the false positives are common. The contamination is mainly caused by carry-over from previous PCR products and the cross-contamination among samples and reagents. Therefore, in clinical NAAT, the blank control, the negative sample control and reagents control should be included. In pooled NAAT, the negative pooled control and positive pooled controls should be added in order to control the error associated with a manual sample mixing process. However the false positive cannot be avoided completely. Thus, the possibility of false positives is a disadvantage of NAAT.

In in the observation period of this open prospective cohort study, 114 established HIV infections and 53 acute/recent HIV infections were detected. If only ELISA and WB were used, 29 antibody negative or indeterminate acute HIV infections would fail to be diagnosed in a timely fashion. If NAAT was also applied for diagnosis, the cost of detecting an acute HIV infection was $998, which is close to 1.5 times of the cost of an acute infection diagnosis with antibody targeting assay only. Furthermore, the cost of detecting a window period HIV infection reached $3618. Although much less than the cost in public health settings of the United States [25], the cost of pooled NAAT was high even in high-risk populations with high HIV incidences. In this study, there were several major factors affecting cost. First, to control the potential contamination, the negative control, low positive control, high positive control, negative pooled control and positive pooled control were included in each of the 24 assays, which increased the total cost by 38.9%. Second, because of the high mobility and limited compliance of this MSM high risk population, follow-up rate of this cohort reached just 59.8%. Some potential HIV seroconverted cases could not be reached for follow-up, which may have impacted the acute HIV infection detection rate. Therefore, if the follow-up rate could be increased, the costs of acute and window period HIV infections diagnosis would be lower. Third, in this study, the widely used third generation ELISA HIV antibody assay was adopted instead of the commercialized fourth generation HIV ELISA assays, even though the fourth HIV ELISA shortens the window period of serologic assays [18], [32]. In our study, 6 of the 8 window period positive samples were detected with fourth generation ELISA, and 4 of which were proved to be positive, although they were not presented in our results. Therefore, if the fourth generation ELISA assay was used in this whole study, more acute HIV infections would be detected with serologic assays,which would greatly reduce the likelihood of utilizing the pool splitting process, thus reducing the cost of NAAT. Furthermore, for the cost of commercial fourth generation ELISA is similar to third generation ELISA in China, and so using fourth generation ELISA in serologic screening would not produce more cost itself, but the frequency and cost on the confirmation of the HIV antigen would need to be evaluated. The cost of pooled NAAT varied greatly among other studies abroad depending on the target population and detection strategy, but the cost determined in this study was generally lower than that of other studies [15], [28].

It is reported that the viral load in acute infection stage is at its peak level of the disease process [31], and the transmission efficiency is much higher than in chronic HIV infections and Acquired Immune Deficiency Syndrome (AIDS) patients [33], [34], [35]. Therefore, an acute HIV infection has a higher dissemination risk from a public health perspective. Pooled NAAT can improve the detection rate of acute HIV infections significantly. By informing patients of their HIV status, promoting knowledge of AIDS prevention, teaching patients how to take effective protective measures, and treating them with the appropriate drugs, we can reduce the transmission ability of the acutely infected HIV patients considerably. Since 2002, HIV infected patients can receive HAART treatment freely under the “four free and one care” policy in China. Based on the current first-line antiretroviral regimen, the Chinese government will spend about $781 on treatment, CD4 count, and viral load monitoring for each HIV infected case per year. Obviously, this is a heavy economic burden. In this study, through the actual expenses of the HIV screening assay and the calculated second generation HIV transmissions averted using previously published parameters of the MSM population. we found the addition of pooled NAAT after third-generation ELISA had a cost-effectiveness ratio of $3360 per gained QALY, which is far below the levels of the general population and MSM high-risk populations abroad [25], [36], [37]. The input-output ratio was 1∶16.9. Some studies have shown that utilizing pooled NAAT after HIV antibody assay was not cost-effective in populations with low HIV incidences [25], [37]. However, this study demonstrated that, for a high-risk population with an HIV incidence as high as 7.1/100person years, this strategy could prevent a much larger subsequent expense on treatment and monitoring in the long run.

In conclusion, the addition of the pooled NAAT to the current screening strategy of third generation ELISA HIV antibody test and WB tests in high risk populations at voluntary counseling & testing centers can help to improve early diagnosis of acute HIV infections, especially within window period cases Further improvement may be seen if fourth generation ELISA is adopted. This strategy will assist in controlling HIV transmission, allow long-term savings in government funds allocated to treating and preventing HIV/AIDS, and ultimately help to fight HIV/AIDS more efficiently, effectively, and economically.

## Materials and Methods

### Ethics statement and study subjects

An open prospective cohort of MSM was recruited by the categorical snowball sampling method in three cities of Liaoning Province: Shenyang, Anshan and Fushun, between February 2009 and September 2010. All consenting persons who presented for HIV counseling and testing at Voluntary Counseling and Testing sites in these 3 cities, volunteered to participate in this study and met the following criteria were included. The criteria included: male, above 18 years of age, having at least one male sexual partner in the past 12 months, and physically able. All cases in the MSM high-risk cohort provided written informed consent for the collection of samples and subsequent analyses, and completed a questionnaire administered by trained interviewers. Interviews were conducted and blood samples were obtained and tested for HIV at follow-up visits every 10 weeks. All participants were given general information about HIV and were informed how to practice safe sexual behaviors during the course of the pre- and post-test counseling provided by this study. All HIV positive participants were informed to bring their male sex partners to the testing site to be tested for HIV. This study was approved by the Medical Research Ethics Committee of No. 1 Hospital of China Medical University and written informed consent was obtained from all participants.

### HIV Antibody Screening

**Figure 2:**
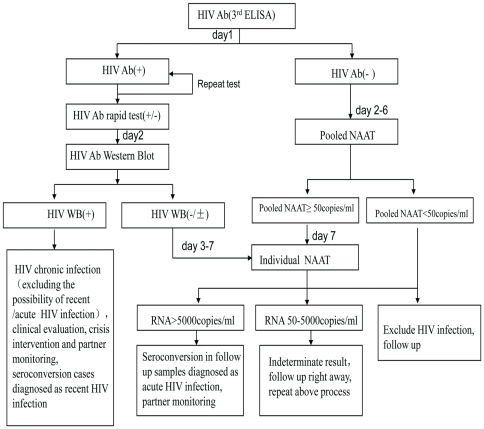
The Algorithm of the Procedures of the MSM Voluntary Counseling & Testing centers for HIV Testing and Diagnosis. HIV detection procedure, detecting time, algorithm of HIV-1 antibody screening and Pooled NAAT process, diagnostic criteria and principles of management are shown in the flow chart.

EDTA anticoagulant whole blood was collected in sterile condition. Plasma was separated within 6 hours. All of the plasma was tested with a third-generation HIV Antibody ELISA test, Vironostika HIV-1/2 Microelisa System, (BioMérieux, Holland) within 24 hours according to the manufacturer's instructions. Negative samples were tested with pooled NAAT. Positive samples were retested using the same ELISA test and an additional rapid test, Determine HIV 1/2 (Abbot, USA). Once the samples were determined to be both positive or to contain at least one positive result, HIV status was confirmed with western blot assay within 24 hours. If the results were both negative, the plasma was detected with pooled NAAT within one week (Figure 2).

### Western Blot assays (WB)

The HIV-1/2 western blot assay HIV Blot 2.2 WB (Genelabs Diagnostics, Singapore) was used for confirming HIV-1 infection. Negative, weak positive and strong positive controls were included each time according to the manufacturer's instructions. The participants were divided into 3 groups (chronic HIV infection, acute HIV infection and recent HIV infection) according to the national HIV detection guidelines. In brief, chronic HIV infection was diagnosed if all HIV-1 specific bands were detected in the WB and the possibility of recent HIV infection was excluded based upon the high-risk behavior information in the epidemiology survey. Acute HIV infection was diagnosed if the HIV-1 specific bands were not up to the positive standard, yet demonstrated an increasing trend in the follow-up blood samples. Recent HIV infection was characterized if the plasma became HIV-1 positive during the follow-up visits. Recent and acute HIV infections were both included in the calculation of HIV incidence density. The negative or indeterminate samples in WB were detected with NAAT individually without mixing.

### Pooled or Individual NAAT

**Figure 3:**
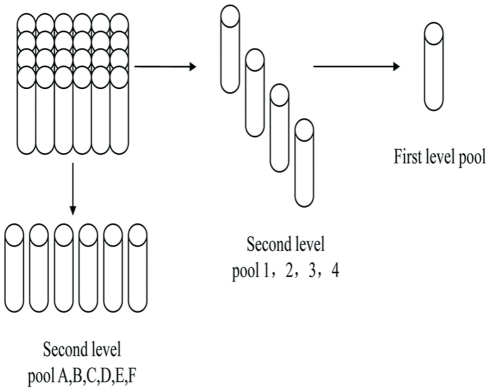
24 Mini Pool Mix and Split Process. Every 6 samples were mixed to 4 crosswise second level pools, every 4 samples were mixed to 6 lengthways second level pools, and the 4 crosswise second level pools were mixed to a first level pool. If the first level pool was positive, the 10 second level pools were detected at the same time.

According to the follow-up frequency and daily number of clinical patients in our cohort, a 24-sample mini-pool (6×4) strategy was adopted. Every 6 samples were mixed to 4 crosswise second level pools, every 4 samples were mixed to 6 lengthways second level pools, and the 4 crosswise second level pools were mixed to a first level pool (Figure 3). A commercial HIV RNA quantitative detection assay, COBAS AmpliPrep/COBAS TaqMan HIV-1 Test (Roche, Germany), was used in HIV-1 NAAT. If the first level pool was positive, the 10 second level pools were detected at the same time in the following day to validate the positive sample. Negative control, weak positive control, strong positive control, negative pool control, and positive pool control were used in the pooled NAAT each time. Samples with positive ELISA but negative or indeterminate WB results were subjected to NAAT directly without mixing. All NAAT positive cases were followed-up. If a follow-up sample became positive in WB, the case was diagnosed as a window period acute HIV infection and included in the calculation for HIV incidence. Participants without seroconversion were followed-up continually according to the previous schedule and participants with RNA copy number above 5000 copies/ml were followed-up until they were confirmed with WB. Participants with RNA copy number between 50–5000 copies/ml were reexamined with newly collected blood. NAAT negative samples were also followed according to the previous follow-up schedule. The algorithms of the detection and follow-up procedures are shown in Figure 2.

### Gained quality-adjusted life years (QALYs) and Averted Lifetime Medical Costs by Pooled NAAT Screening for AHI

We evaluated QALYs gained from transmissions averted and the input-output ratio for pooled NAAT after third generation ELISA test. To describe secondary HIV transmission (table 2), we used the basic reproductive number, R_0_. R_0_ can be interpreted as the lifetime number of subsequent infections, attributable to a single infected individual in a susceptible population. According to a mathematical model using a sex-role-preference framework to predict HIV infection in the MSM population [38], each HIV-infected MSM may on average transmit HIV to 3.8 persons during the window period without any behavioral intervention and therapy. In this study we estimated a 25% transmission possibility in the acute HIV infection stage based on methods mentioned in a previous report [39]. The transmission rates for people unaware of their HIV serostatus were reported to be 3.5 times higher than those who are aware of their HIV status [40]. The QALYs gained were expressed as the difference between QALYs for a partner with and without HIV infection. We designated an uninfected person with a QALY of 1.0. Partners were estimated to become infected at the age of 33 based on the average age of AHI in this cohort. According to the World Health Report 2006, Chinese males have an average life span of 72 years. The additional life expectancy for HIV-1 patients treated with HAART (Highly Active Anti-Retroviral Therapy) was 32 years [41], compared to 39 years for healthy people. The acute HIV infection diagnosis can avert 7 QALYs lost in every HIV transmission. Cost-utility estimations were calculated by subtracting projected HIV treatment costs for HIV infections averted from total AHI program costs and dividing by the QALYs gained for each averted HIV infection.
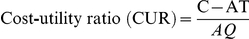


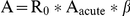



**Table 2:** Explanation of Abbreviation Symbol.

Abbreviation Symbol	Explanatory Note
R_0_	the basic reproduction number for HIV transmission among Chinese MSM.
A_acute_	transmission that might occur in acute/recent HIV infection stage
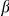	decreased HIV transmission risk after knowing HIV infection status. (3.5−1)/3.5
T	expense for treatment and monitoring life expectancy of second generation transmitted HIV infected patients
Q	QALYs lost due to HIV infection for each patient
